# PW02-041 - Canakinumab treatment regimens in CAPS-patients

**DOI:** 10.1186/1546-0096-11-S1-A182

**Published:** 2013-11-08

**Authors:** F Hofer, T Endres, B Kortus-Götze, N Blank, E Weißbarth-Riedel, C Schuetz, T Kallinich, K Krause, C Rietschel, G Horneff, J Kuemmerle-Deschner

**Affiliations:** 1 Department of Pediatrics, Division of Pediatric Rheumatology, University Hospital Tuebingen, Tuebingen, Germany; 2 Klinik für Innere Medizin, Schwerpunkt Nephrologie, Universitätsklinikum Marburg, Marburg, Germany; 3 Hämatologie, Onkologie u. Rheumatologie, Universitätsklinikum Heidelberg, Heidelberg, Germany; 4 Kinderrheumatologische Ambulanz, Universitätsklinikum Eppendorf, Hamburg, Germany; 5 Klinik für Kinder und Jugendmedizin, Universitätsklinikum Ulm, Ulm, Germany; 6 Kinderklinik Sektion Rheumatologie, Charité Campus Virchow, Germany; 7 „Allergie-Centrum Charité“, Klinik für Dermatologie, Charité Campus Mitte, Berlin, Germany; 8 Rheumatologische Ambulanz , Clementine Kinderhospital, Frankfurt, Germany; 9 Abteilung für. Allgemeine Kinder- und Jugendmedizin, Asklepios Klinik Sankt Augustin, St. Augustin, Germany

## Introduction

Canakinumab is a recombinant monoclonal fully human antibody against Interleukin-1β and currently the only drug approved for the treatment of CAPS in Europe. Current dose recommendations are 150mg (body weight >40kg) respectively 2mg/kg bodyweight (15 to 40kg) every 8 weeks but yield insufficient response in some individuals, especially in children and patients with severe phenotypes [1].

## Objectives

In this study we analyzed the response to daily practice (in contrast to trial condition) canakinumab treatment regimens in CAPS-patients with focus on age, mutation and clinical presentation and the necessity and effect of dose adjustment.

## Methods

An observational national multicenter study was conducted. CAPS-Patients were included if they received at least two doses of canakinumab. Data included information regarding demographics, treatment, clinical disease activity and inflammatory markers (including SAA, CRP, S100, ESR, IL-6). Response to treatment was assessed using CAPS-disease activity scores, CRP and/or SAA levels.

## Results

A cohort of 68 patients with CAPS was analyzed. At the beginning of treatment 27 patients had been younger than 18 years with a median age of 25.4 years (range 22 months to 73 years). The most frequent mutations were R260W, A439V, E311K, V198M, Q703K and most patients showed MWS or FCAS/MWS phenotype (3 patients with NOMID, 4 with MWS/NOMID). The median treatment duration was 855 days (range: 28-1973 days). In 57% (39) of patients full response was sustained until next scheduled drug application (34% (23) partial remission). With standard treatment 31% (21) of patients achieved full response. In 44% (30) of all patients canakinumab dose and/or application interval was increased above the standard regimen (2/3 NOMID, 3/4 MWS/NOMID). Two serious adverse events were reported (severe infection, osteonecrosis), mild and moderate adverse events were mostly upper respiratory tract infections but almost no injection site reactions.

## Conclusion

Most CAPS-Patients achieve full remission with canakinumab. However, almost 50% of patients, particularly children, require dose adjustment. Dose increase was well tolerated and full remission was achieved without an increased rate of adverse events.

## Competing interests

F. Hofer: None declared, T. Endres: None declared, B. Kortus-Götze: None declared, N. Blank: None declared, E. Weißbarth-Riedel: None declared, C. Schuetz: None declared, T. Kallinich: None declared, K. Krause: None declared, C. Rietschel : None declared, G. Horneff: None declared, J. Kuemmerle-Deschner Grant / Research Support from: NOVARTIS, Consultant for: NOVARTIS
